# Supplementary material for: Detection of Circulating Tumor Cells in the Diagnostic Leukapheresis Product of Non-Small-Cell Lung Cancer Patients Comparing CellSearch® and ISET
Source: Cancers (Basel). 2020 Apr 7;12(4):896. doi: 10.3390/cancers12040896 (PMC7226321; doi:10.3390/cancers12040896)
Supplement: Supplementary file 1 [file cancers-12-00896-s001.pdf]

Supplementary Materials

Detection of Circulating Tumor Cells in the Diagnostic Leukapheresis Product of Non-Small Cell Lung Cancer Patients Comparing CellSearch® and ISET®

Menno Tamminga, Kiki C. Andree, T.Jeroen N. Hiltermann, Maximilien Jayat, E. Schuuring, Hilda van den Bos, Diana C.J. Spierings, Peter M. Lansdorp, Wim Timens, Leon W.M.M. Terstappen and Harry J.M. Groen

Table S1. Blood cell counts in peripheral blood and DLA product.

Cell counts		Peripheral Blood		DLA Product	
**Blood cells**	**Unit**	**Mean**	**SD**	**Mean**	**SD**
Red blood cells	$\times 10^{12}$/L	4.5	0.6	0.6	0.28
Leukocytes	$\times 10^{9}$/L	10.1	3.8	135	76
Lymphocytes	$\times 10^{9}$/L	1.7	0.8	51.8	22
Monocytes	$\times 10^{9}$/L	0.9	0.4	29	10
Granulocytes	$\times 10^{9}$/L	7.4	3.3	62	30
Platelets	$\times 10^{9}$/L	298	127	1540	377
Hemoglobin	Mmol/L	8.2	1.3	0.91	0.28
Hematocrit	%	41	5.6	0.07	0.04

Table S2. Mean cell counts (pre and post apheresis) in blood and in diagnostic leukaphersesis product.

Blood cells	Unit	Blood Pre DLA	DLA	Blood Post DLA
Red blood cells	$\times 10^{12}$	4.6	0,5	4.2
Leukocytes	$\times 10^{9}$	10.1	133	8.5
Lymfocytes	$\times 10^{9}$	1.7	47	1.3
Monocytes	$\times 10^{9}$	0.9	31	0.6
Granulocytes	$\times 10^{9}$	7.3	69	6
Platelets	$\times 10^{9}$	281	2375	238
Hemoglobin	Mmol/L	8.1	0,9	7.4
Hematocrit	%	40.4	4	37.1

© 2020 by the authors. Licensee MDPI, Basel, Switzerland. This article is an open access article distributed under the terms and conditions of the Creative Commons Attribution (CC BY) license (http://creativecommons.org/licenses/by/4.0/).
